# Hidden loss to follow-up among tuberculosis patients managed by public–private mix institutions in South Korea

**DOI:** 10.1038/s41598-022-16441-7

**Published:** 2022-07-20

**Authors:** Hyung Woo Kim, Sohee Park, Jinsoo Min, Jiyu Sun, Ah Young Shin, Jick Hwan Ha, Jae Seuk Park, Sung-Soon Lee, Marc Lipman, Ibrahim Abubakar, Helen R. Stagg, Ju Sang Kim

**Affiliations:** 1 Division of Pulmonary and Critical Care Medicine, Department of Internal Medicine, Incheon St. Mary’s Hospital, College of Medicine, The Catholic University of Korea, Seoul, Republic of Korea; 2 Institute of Health Services Research, Yonsei University, Seoul, Republic of Korea; 3 Department of Biostatistics, Graduate School of Public Health, Yonsei University, Seoul, Republic of Korea; 4 Division of Pulmonary and Critical Care Medicine, Department of Internal Medicine, Seoul St. Mary’s Hospital, College of Medicine, The Catholic University of Korea, Seoul, Republic of Korea; 5 Division of Biostatistics, Department of Biomedical Systems Informatics, Yonsei University College of Medicine, Seoul, Republic of Korea; 6 Division of Pulmonary Medicine, Department of Internal Medicine, Dankook University College of Medicine, Cheonan, Republic of Korea; 7 Division of Pulmonary and Critical Care Medicine, Department of Internal Medicine, Ilsan Paik Hospital, Inje University College of Medicine, Goyang, Republic of Korea; 8 UCL-TB, University College London, London, UK; 9 Division of Medicine, UCL Respiratory, University College London, London, UK; 10 Royal Free London NHS Foundation Trust, London, UK; 11 Institute for Global Health, University College London, London, UK; 12 Usher Institute, The University of Edinburgh, Edinburgh, UK

**Keywords:** Tuberculosis, Public health

## Abstract

In South Korea, public–private mix (PPM) was launched in 2011. This retrospective cohort study sought to determine the rate of loss to follow-up (LTFU) among drug-susceptible tuberculosis (DS-TB) patients in all nationwide PPM institutions, and the risk factors for LTFU. National notification data for DS-TB patients diagnosed between August 2011 and July 2014 in PPM institutions were analysed. Determination of LTFU included detection of instances where patients were transferred out, but when they did not attend at other TB centres in the following two months. Univariable and multivariable competing risk models were used to determine risk factors for LTFU. 73,046 patients with 78,485 records were enrolled. Nominally, 3426 (4.4%) of records were LTFU. However, after linking the multiple records in each patient, the percentage of LTFU was 12.3% (9004/73,046). Risk factors for LTFU were: being foreign-born (3.13 (95% CI 2.77–3.53)), prior LTFU (2.31 (2.06–2.59)) and greater distance between the patient’s home and the TB centre (4.27 (4.03–4.53)). ‘Transfer-out’ was a risk factor in patients managed by treatment centres close to home (1.65 (1.49–1.83)), but protective for those attending centres further (0.77 (0.66–0.89)) or far-away (0.52 (0.46–0.59)) from home. By considering the complete picture of a patient’s interactions with healthcare, we identified a much higher level of LTFU than previously documented. This has implications for how outcomes of treatment are reported and argues for a joined-up national approach for the management and surveillance of TB patients, in nations with similar healthcare systems.

## Introduction

South Korea is an ‘intermediate’ tuberculosis (TB) incidence country. Since the Korean War, and with increased economic growth, its TB burden has fallen^1^. In the early 1990s TB incidence in South Korea was 202 per 100,000 population^2^, which decreased by half within the following decade. Such improvements were in part due to better access to high-quality healthcare; National Health Insurance (NHI) was enacted in 1963 and coverage extended to the majority of the population by 1989^3^. It is now characterized as universal population coverage with a single-payer system since 2000^4^. However, the rate of decline in TB incidence slowed during the 2000s such that the incidence of TB was similar in 2001 and 2011, at 96.3 cases per 100,000, and 100.8 cases per 100,000 population, respectively^5^.

Patients in South Korea can attend any hospital nationwide with the financial support of NHI^6^. Approximately 90% of healthcare facilities are private, with the role of public healthcare centres in provision of curative services being very little^7,8^. In 2011, public healthcare centres accounted for only 2.6% of out-patient visits, which was lower in metropolitan areas (1.3%)^9^. As a result, the proportion of TB patients receiving treatment in the private sector has increased year on year such that in 2001 and 2011, 53.9% and 88.7% of the national notified TB cases were reported from private hospitals^5^.

The stagnation in decline of TB incidence after 2000 was thought to result from a low treatment success rate in the private sector^10,11^. Only 75% of patients achieved treatment success in private hospitals in the early 2000s due to a high percentage (11.6%) of lost to follow-up (LTFU). This compared to only 2.5% in the public sector^10^. As a result, in 2011, the government of South Korea launched a public–private mix (PPM) project for TB control, as recommended by the World Health Organization (WHO)^12^. In 2016, a total of 128 PPM hospitals from across the country participated in the PPM project, accounting for an estimated 65% of all national TB patients. In 2020, 77.4% of total TB patients in South Korea were notified and managed at 164 nationwide PPM hospitals^13^.

After implementation of the PPM project, treatment success among sputum smear-positive pulmonary TB patients increased from 68.0% in 2011 to 88.3% in 2016^14^. TB incidence in South Korea, which had been stagnant within the range of 80 and 100 cases per 100,000 population, firstly decreased below the level of 80 cases per 100,000 population in 2016 (76.8 cases per 100,000 population). TB incidence abruptly decreased thereafter—that in 2020 was 49.4 cases per 100,000 population.

As LTFU lead to prolonged infectiousness, relapse, death, acquired drug resistance and treatment failure^15^, reducing LTFU is important in national tuberculosis control. Previously, only small hospital-based or city-wide studies have identified risk factors for LTFU in South Korea^16,17^. Here we report a retrospective cohort study of drug-susceptible TB (DS-TB) patients notified in PPM institutions across the country, designed to estimate the frequency of, and risk factors for LTFU. Our cohort represent TB patients managed at private sectors, between 2011 and 2014. By focusing on this period, we could identify the problem of private sectors at early stage of PPM project introduction which would facilitate investigating the factor that contributed to the decrease in TB burden. In addition, our study uses more sophisticated methodologies to determine LTFU than previously, by taking into account the full picture of a patient’s interactions (or absence of interactions) with healthcare systems across their treatment course.

## Results

### Characteristics of the treatment cohort

**Figure 1:**
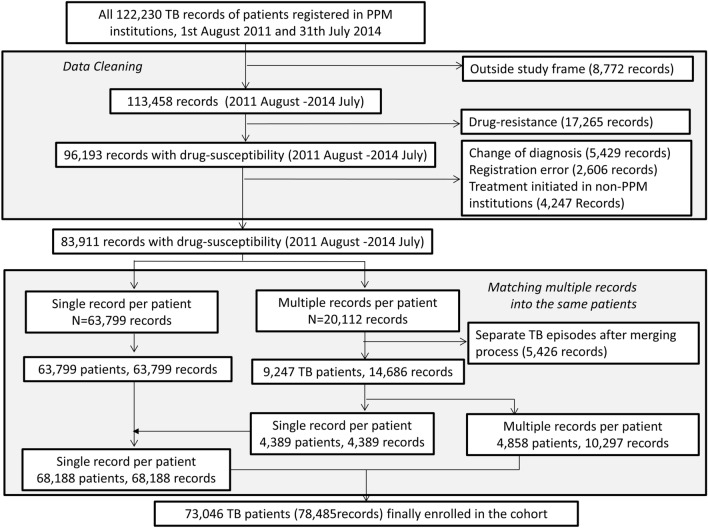
Patient enrolment flow chart. After applying exclusion criteria, 83,911 records were classified into those with a single notification per patient and those with multiple notifications. After merging the records of the latter into the one outcome, a total of 73,046 patients (78,485 records) were finally enrolled in this study. *TB* tuberculosis, *PPM* public–private mix.

After applying our inclusion and exclusion criteria, data on a total of 73,046 patients with 78,485 records were available from the Korean National TB Surveillance System (KNTSS) (Fig. 1). The total follow-up time was 39,206.0 person-years. 68,188 patients had a single record and 4,858 patients had multiple records (Table 1). Of 73,046 patients with DS-TB, 41,756 (57.2%) were male, and 1,183 (1.6%) foreign-born (Table 2). The median age of all patients was 54 (interquartile range 37–71) years. More than 90% had pulmonary involvement, and over 80% had no history of prior treatment for TB. The majority (81.7%) of patients lived in the same district as the medical institution where they were treated (Table 3).

**Table 1:** Treatment outcomes for tuberculosis patients at before and after the process of merging and reclassifying records.

Categories of treatment outcome	All TB patients		Single-record group		Multiple-record group	
	Records before the process (N = 78,485)	Patients after the process (N = 73,046)	Records before the process (N = 68,188)	Patients after the process (N = 68,188)	Records before the process (N = 10,297)	Patients after the process (N = 4,858)
Treatment success	58,347 (74.3)	48,136 (65.9)	53,362 (78.3)	45,487 (66.7)	4,985 (48.4)	2,649 (54.5)
Treatment failed	86 (0.1)	35 (0.0)	78 (0.1)	31 (0.0)	8 (0.1)	4 (0.1)
Loss to follow-up	3,426 (4.4)	9,004 (12.3)	2,995 (4.4)	8,118 (11.9)	431 (4.2)	886 (18.2)
**Transfer-out**					–	
No further registration	5,304 (6.8)	–	4,609 (6.8)	–	695 (6.7)	–
Re-registration ≤ 60 days	2,761 (3.5)	–	668 (1.0)	–	2,093 (20.3)	–
Re-registration > 60 days	2,511 (3.2)	–	1,449 (2.1)	–	1,062 (10.3)	–
Died	4,563 (5.8)	4,241 (5.8)	4,060 (6.0)	3,906 (5.7)	503 (4.9)	335 (6.9)
Other	299 (0.4)	290 (0.4)	232 (0.3)	240 (0.4)	67 (0.7)	50 (1.0)
Still on treatment	736 (0.9)	11,340 (15.5)	735 (1.1)	10,406 (15.3)	1 (0.0)	934 (19.2)
Diagnosis changed	452 (0.6)	–	–	–	452 (4.4)	–

Data are presented as n (%).

*TB* tuberculosis.

**Table 2:** Baseline demographic characteristics of enrolled tuberculosis patients, categorized by treatment outcome.

Variables	Loss to follow-up	Treatment success	Treatment failed	Death	Other	Still on treatment	Total
Total N (row %)	9,004 (12.3)	48,136 (65.9)	35 (0.0)	4,241 (5.8)	290 (0.4)	11,340 (15.5)	73,046 (100.0)
**Gender**							
Male	5,481 (60.9)	26,578 (55.2)	30 (85.7)	2,850 (67.2)	189 (65.2)	6,628 (58.4)	41,756 (57.2)
Female	3,523 (39.1)	21,558 (44.8)	5 (14.3)	1,391 (32.8)	101 (34.8)	4,712 (41.6)	31,290 (42.8)
**Age groups (years)**							
0–19	222 (2.5)	2,018 (4.2)	1 (2.9)	4 (0.1)	5 (1.7)	356 (3.1)	2,606 (3.6)
20–34	1,345 (14.9)	9,832 (20.4)	9 (25.7)	43 (1.0)	46 (15.9)	2,140 (18.9)	13,415 (18.4)
35–49	1,540 (17.1)	10,329 (21.5)	10 (28.6)	249 (5.9)	58 (20.0)	2,595 (22.9)	14,781 (20.2)
50–64	1,968 (21.9)	11,297 (23.5)	13 (37.1)	703 (16.6)	66 (22.8)	2,882 (25.4)	16,929 (23.2)
65 or above	3,929 (43.6)	14,660 (30.5)	2 (5.7)	3,242 (76.4)	115 (39.7)	3,367 (29.7)	25,315 (34.7)
**Nationality**							
Native patients	8,706 (96.7)	47,454 (98.6)	35 (100.0)	4,226 (99.6)	280 (96.6)	11,162 (98.4)	71,863 (98.4)
Foreign-born patients	298 (3.3)	682 (1.4)	0 (0.0)	15 (0.4)	10 (3.4)	178 (1.6)	1,183 (1.6)
**Place of residence**							
Urban	8,850 (98.3)	47,456 (98.6)	35 (100.0)	4,190 (98.8)	288 (99.3)	11,224 (99.0)	72,043 (98.6)
Rural	154 (1.7)	680 (1.4)	0 (0.0)	51 (1.2)	2 (0.7)	116 (1.0)	1,003 (1.4)

^a^Composed of ‘treatment after failure patients’ and ‘other previously treated patients’ whose outcome of previous treatment was unknown or undocumented. ^b^Patients with pulmonary tuberculosis were analysed. *N* number, *LTFU* loss to follow-up; Data are presented as n (column %).

**Table 3:** Clinical and Treatment related characteristics of enrolled tuberculosis patients, categorized by treatment outcome.

Variables	Loss to follow-up	Treatment success	Treatment failed	Death	Other	Still on treatment	Total
Total N (row %)	9,004 (12.3)	48,136 (65.9)	35 (0.0)	4,241 (5.8)	290 (0.4)	11,340 (15.5)	73,046 (100.0)
**Previous TB treatment history**							
New patients	7,063 (78.4)	41,393 (86.0)	24 (68.6)	3,453 (81.4)	212 (73.1)	8,758 (77.2)	60,903 (83.4)
Treatment after LTFU	313 (3.5)	440 (0.9)	3 (8.6)	73 (1.7)	14 (4.8)	272 (2.4)	1,115 (1.5)
Relapse	1,034 (11.5)	4,349 (9.0)	6 (17.1)	523 (12.3)	33 (11.4)	1,763 (15.5)	7,708 (10.6)
Other previously treated patients^a^	594 (6.6)	1,954 (4.1)	2 (5.7)	192 (4.5)	31 (10.7)	547 (4.8)	3,320 (4.5)
**Location of TB**							
PTB only	6,403 (71.1)	32,180 (66.9)	29 (82.9)	3,353 (79.1)	190 (65.5)	7,250 (63.9)	49,405 (67.6)
EPTB only	552 (6.1)	3,480 (7.2)	1 (2.9)	152 (3.6)	19 (6.6)	920 (8.1)	5,124 (7.0)
Both PTB and EPTB	2,049 (22.8)	12,476 (25.9)	5 (14.3)	736 (17.4)	81 (27.9)	3,170 (28.0)	18,517 (25.3)
**Chest X-ray**^**b**^							
Suspicious TB lesions	7280 (86.1)	38,398 (86.0)	29 (85.3)	3403 (83.2)	212 (78.2)	8661 (83.1)	57,983 (85.4)
Normal	157 (1.9)	1051 (2.4)	0 (0.0)	64 (1.6)	10 (3.7)	349 (3.3)	1631 (2.4)
Unknown	216 (2.6)	1176 (2.6)	1 (2.9)	169 (4.1)	8 (3.0)	264 (2.5)	1834 (2.7)
Not done	799 (9.5)	4031 (9.0)	4 (11.8)	453 (11.1)	41 (15.1)	1146 (11.0)	6474 (9.5)
**Baseline sputum AFB smear test**^**b**^							
Smear positive	2837 (33.6)	13,120 (29.4)	20 (58.8)	2128 (52.0)	72 (26.6)	3881 (37.2)	22,058 (32.5)
Smear negative	4389 (51.9)	25,925 (58.1)	10 (29.4)	1640 (40.1)	142 (52.4)	4864 (46.7)	36,970 (54.4)
Unknown	1226 (14.5)	5611 (12.6)	4 (11.8)	321 (7.9)	57 (21.0)	1675 (16.1)	8894 (13.1)
**Distance from home to treatment centre**							
Same district (close)	5,357 (59.5)	40,934 (85.0)	21 (60.0)	3,517 (82.9)	223 (76.9)	9,661 (85.2)	59,713 (81.7)
Neighbouring district (far)	1,761 (19.6)	4,270 (8.9)	8 (22.9)	400 (9.4)	28 (9.7)	915 (8.1)	7,382 (10.1)
Far-away district (far-away)	1,886 (20.9)	2,932 (6.1)	6 (17.1)	324 (7.6)	39 (13.4)	764 (6.7)	5,951 (8.1)
**Number of TB notification records**							
A single record	8,118 (90.2)	45,487 (94.5)	31 (88.6)	3,906 (92.1)	240 (82.8)	10,406 (91.8)	68,188 (93.3)
Multiple records	886 (9.8)	2,649 (5.5)	4 (11.4)	335 (7.9)	50 (17.2)	934 (8.2)	4,858 (6.7)
**Duration of anti-TB treatment**							
Median (Range)	58 (0–300)	189 (166–300)	213 (124–291)	39 (0–300)	45 (0–299)	300 (0–300)	189 (0–300)
Mean (± SD)	79.1 (± 71)	210.5 (± 39.1)	220.1 (± 50.6)	64.7 (± 67.4)	73.4 (± 77.1)	278.7 (± 65.5)	195.9 (± 80.8)

^a^Composed of ‘treatment after failure patients’ and ‘other previously treated patients’ whose outcome of previous treatment was unknown or undocumented. ^b^Patients with pulmonary tuberculosis were analysed. *N* number, *LTFU* loss to follow-up, *TB* tuberculosis, *PTB* pulmonary tuberculosis, *EPTB* extra-pulmonary tuberculosis, *SD* standard deviation, *AFB* acid-fast bacillus. Data are presented as n (column %).

### Treatment outcomes, focussing on losses to follow-up

Before the process of merging and reclassification, treatment success (cure and treatment completed) was reported in 74.3% of cases (Table 1). 3,426 (4.4%) cases were initially reported as LTFU. However, there were 5,304 (6.8%) records with no further registration after transfer-out and 2,511 (3.2%) where re-registration was 61 days or more after transfer-out; most were re-categorized as LTFU. Thus, the percentage LTFU increased from 4.4 to 12.3% after the merging and reclassification processes. Among all TB patients, the number of cases with an outcome of death or treatment failure were 4,241 (5.8%) and 35 (< 0.1%), respectively.

The median duration of treatment was 189 days (range 0–300) for all patients. Among individuals who were LTFU this was 58 days (range 0–300), with 4,597 (51.1%) becoming LTFU during the intensive and 4407 (48.9%) during the continuation phase.

### Risk factors associated with losses to follow up

Risk factors for LTFU among all included TB patients were investigated using univariable Fine and Gray models (Table 4). Within the cohort, the overall rate of LTFU was 229.7 per 1,000 person years. Females (hazard ratio (HR): 0.85, (95% confidence interval: 0.81–0.88), p < 0.001) showed a lower rate of LTFU. When compared with patients aged < 20 years, age groups 20–34 (HR: 1.18 (1.02–1.37), p = 0.023), 35–49 (HR: 1.24 (1.07–1.43), p = 0.003), 50–64 (HR: 1.40 (1.22–1.62), p < 0.001), and 65 or above (HR 2.07 (1.80–2.38), p < 0.001) were risk factors for LTFU. Foreign-born patients (HR: 2.20 (1.95–2.47), p < 0.001) and those with multiple notifications (HR: 1.56 (1.46–1.67), p < 0.001) had an increased rate of LTFU. When compared with those with no previous TB history, people treated after previous LTFU (HR: 2.57 (2.30–2.87), p < 0.001) showed an increased rate of LTFU.

**Table 4:** Analysis of risk factors for loss to follow-up (versus all other outcomes) among all tuberculosis patients.

Variables	Total N	Total follow-up (pyrs)	LTFU cases (n)	Rate of LTFU (per 1,000 pyrs)	Univariable analysis HR (95% CI)	Multivariable analysis HR (95% CI)
**Gender**						
Male	41,756	22,256.2	5,481	246.3	1	1
Female	31,290	16,949.8	3,523	207.8	0.85 (0.81–0.88)	0.87 (0.83–0.91)
**Age groups (years)**						
0–19	2,606	1455.9	222	152.5	1	1
20–34	13,415	7631.3	1,345	176.2	1.18 (1.02–1.37)	1.08 (0.93–1.25)
35–49	14,781	8430.7	1,540	182.7	1.24 (1.07–1.43)	1.15 (1.00–1.33)
50–64	16,929	9403.9	1,968	209.3	1.40 (1.22–1.62)	1.28 (1.11–1.48)
65 or above	25,315	12,284.2	3,929	319.8	2.07 (1.80–2.38)	1.93 (1.68–2.21)
**Nationality**						
Native patients	71,863	38,613.5	8,706	225.5	1	1
Foreign-born patients	1,183	592.4	298	503.1	2.20 (1.95–2.47)	3.13 (2.77–3.53)
**Previous TB treatment history**						
New patients	60,903	32,661.3	7,063	216.2	1	1
Treatment after LTFU	1,115	575.5	313	543.8	2.57 (2.30–2.87)	2.31 (2.06–2.59)
Relapse	7,708	4360.7	1,034	237.1	1.13 (1.06–1.21)	1.10 (1.03–1.17)
Other previously treated patients^a^	3,320	1608.4	594	369.3	1.65 (1.51–1.80)	1.38 (1.26–1.51)
**Location of TB**						
PTB only	49,405	26,057.7	6,403	245.7	1	1
EPTB only	5,124	2868.4	552	192.4	0.81 (0.74–0.88)	0.84 (0.77–0.92)
Both PTB and EPTB	18,517	10,279.8	2,049	199.3	0.83 (0.79–0.87)	0.91 (0.86–0.96)
**Number of TB notification records**						
A single record	68,188	36,605.5	8,118	221.8	1	1
Multiple records	4,858	2600.4	886	340.7	1.56 (1.46–1.67)	0.88 (0.82–0.95)
**Place of living**						
Urban	72,043	38,713.4	8,850	228.6	1	1
Rural	1,003	492.6	154	312.7	1.29 (1.10–1.52)	0.70 (0.59–0.83)
**Distance from home to treatment centre**						
At the same district (close)	59,713	33,193.0	5,357	161.4	1	1
Neighbouring district (far)	7,382	3471.0	1,761	507.4	3.03 (2.87–3.20)	3.08 (2.91–3.26)
Far-away district (far-away)	5,951	2542.0	1,886	741.9	4.36 (4.13–4.60)	4.27 (4.03–4.53)

^a^Composed of ‘treatment after failure patients’ and ‘other previously treated patients’ whose outcome of previous treatment was unknown or undocumented. *HR* hazard ratio, *CI* confidence interval, *LTFU* loss to follow-up, *TB* tuberculosis, *PTB* pulmonary tuberculosis, *EPTB* extra-pulmonary tuberculosis.

**Figure 2:**
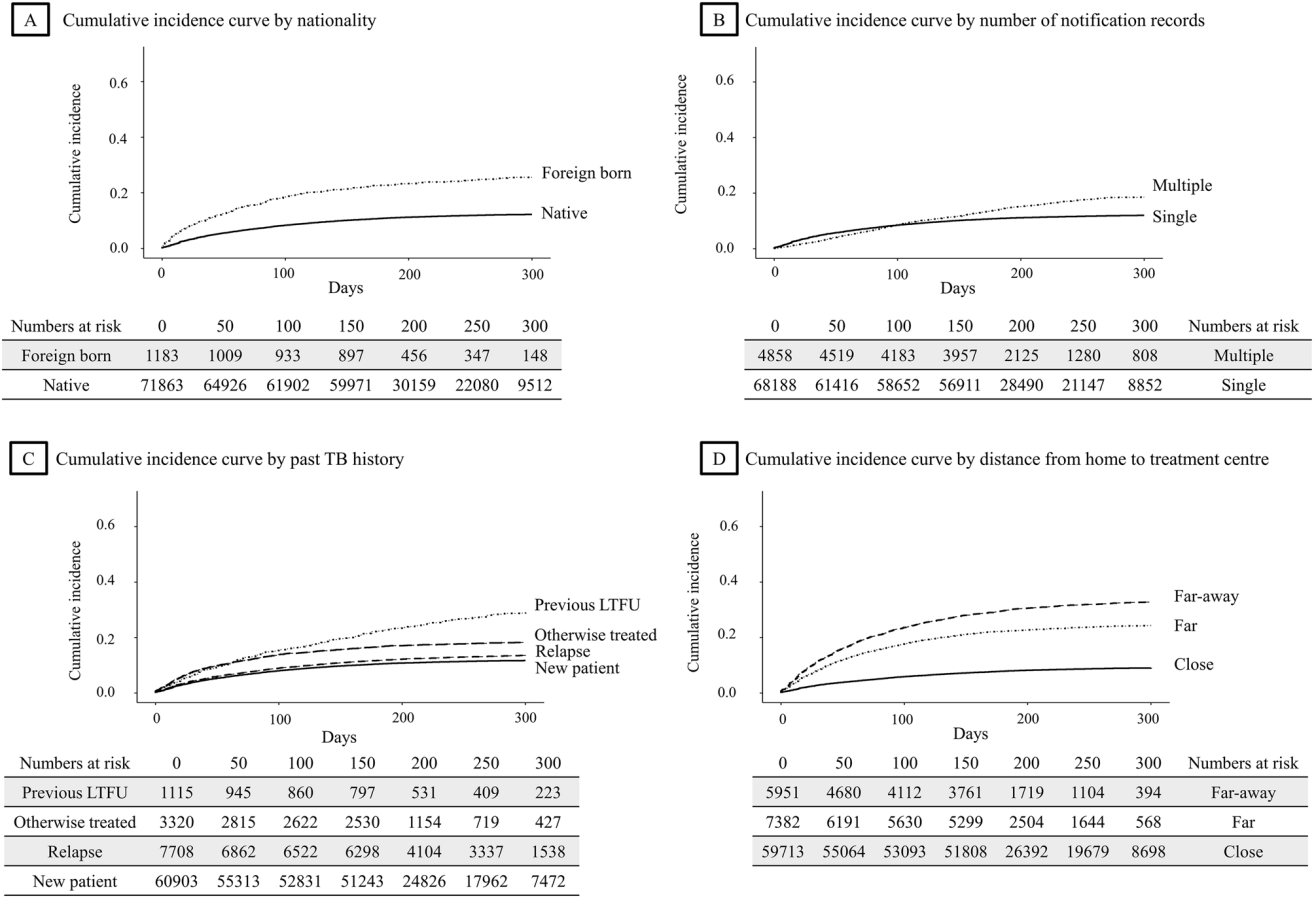
Cumulative incidence curve by nationality, number of notification records, past tuberculosis history and distance from home to treatment centre. *LTFU* loss to follow-up, *TB* tuberculosis. Among the type of past TB history, ‘otherwise treated’ denoted that ‘treatment after failure patients’ and ‘other previously treated patients’ whose outcome of previous treatment was unknown or undocumented. The distance ‘close’ applied to cases where the treatment centre and patient’s residence were within the same municipal level divisions (district, city, or county). ‘Far’ applied to cases where the treatment centre was in the different district, city or county but located within the same large administrative divisions (province or metropolitan city). ‘Far-away’ applied to cases where the treatment centre was located within the different large administrative divisions.

The distance between home and treatment centre was a risk factor for LTFU: compared with patients whose home and treatment centre were located in the same district, those treated in districts far (HR: 3.03 (2.87–3.20), p < 0.001), and far-away from home (HR: 4.36 (4.13–4.60), p < 0.001) had an increased rate. Cumulative incidence curves visualizing the effects of major variables are presented in Fig. 2.

In a multivariable analysis containing all possible risk factors, the effects of most variables were consistent with those in the univariable analysis. However, the direction of association between multiple notifications and LTFU was reversed (HR: 0.88 (0.82–0.95), p = 0.001).

To determine any influence of the distance from home to the treatment centre on the association between transfer-out on LTFU, we tested for modification of the effect of multiple notifications on LTFU by distance (Table 5). When compared with patients with single notification record, the rate of LTFU among patients with multiple notification records was higher (HR: 1.65 (1.49–1.83), p < 0.001) in ‘close’ group, indicating multiple notifications was a risk factor for LTFU among the ‘close’ group. However, in ‘far’ group, the rate of LTFU was lower among the ‘multiple records’ group than in the ‘single record’ group (HR: 0.77 (0.66–0.89), p < 0.001). Likewise, in ‘far-away’ group, LTFU was lower among the ‘multiple records’ group than the ‘single record’ group (HR: 0.52 (0.46–0.59), p < 0.001). These results demonstrated that multiple notifications were a protective factor for LTFU among ‘far’ or ‘far-away’ groups.

**Table 5:** Modification of the effect of transfer-out (multiple records) on LTFU by distance from the patient’s home to treatment centre.

	Single record	Multiple records	RRs (95% CI) for multiple records within strata of distance
	HR (95% CI)	HR (95% CI)	
Close	1	1.65 (1.49–1.83), *P* < 0.001	1.65 (1.49–1.83), *P* < 0.001
Far	3.24 (3.05–3.44), *P* < 0.001	2.49 (2.17–2.86), *P* < 0.001	0.77 (0.66–0.89), *P* < 0.001
Far-away	4.92 (4.63–5.22), *P* < 0.001	2.57 (2.29–2.88), *P* < 0.001	0.52 (0.46–0.59), *P* < 0.001

(1) Effect modification by distance ‘Far’.

Measure of effect modification on additive scale: RERI (95% CI) = − 1.40 (− 1.82–− 0.99); *P* < 0.001.

Measure of effect modification on multiplicative scale: ratio of RRs (95% CI) = 0.47 (0.39–0.56); *P* < 0.001.

(2) Effect modification by distance ‘Far-away’.

Measure of effect modification on additive scale: RERI (95% CI) =  − 3.00 (− 3.43–− 2.57); *P* < 0.001.

Measure of effect modification on multiplicative scale: ratio of RRs (95% CI) = 0.32 (0.27–0.37); *P* < 0.001.

RRs are adjusted for age, gender, nationality, previous TB treatment history, location of TB and place of living.

The ‘single record’ group represents patients who attend one treatment centre during a tuberculosis episode whereas the ‘multiple records’ group indicates those who attend multiple treatment centres (transfer-out). The distance ‘close’ applied to cases where the treatment centre and patient’s residence were within the same municipal level divisions (district, city, or county). ‘Far’ applied to cases where the treatment centre was in the different district, city or county but located within the same large administrative divisions (province or metropolitan city). ‘Far-away’ applied to cases where the treatment centre was located within the different large administrative divisions.

The results of a sensitivity analysis where only TB cases with pulmonary involvement were included in the model were similar to those described above (Table 6).

**Table 6:** Analysis of risk factors for loss to follow-up (versus all other outcomes) among the tuberculosis patients with pulmonary tuberculosis.

Variables	Total N	Total follow-up (pyrs)	LTFU cases (n)	Rate of LTFU (per 1,000 pyrs)	Univariable analysis HR (95% CI)	Multivariable analysis HR (95% CI)
**Gender**						
Male	39,637	21,094.0	5,245	248.6	1	1
Female	28,285	15,243.5	3,207	210.4	0.85 (0.81–0.89)	0.86 (0.82–0.90)
**Age groups (years)**						
0–19	2,424	1351.1	213	157.6	1	1
20–34	12,458	7066.4	1,235	174.8	1.14 (0.98–1.32)	1.04 (0.90–1.21)
35–49	13,591	7729.8	1,451	187.7	1.24 (1.07–1.43)	1.15 (0.99–1.34)
50–64	15,504	8596.1	1,822	212.0	1.38 (1.19–1.59)	1.27 (1.10–1.47)
65 or above	23,945	11,593.9	3,731	321.8	2.02 (1.75–2.33)	1.93 (1.67–2.23)
**Nationality**						
Native patients	66,857	35,814.4	8,172	228.2	1	1
Foreign-born patients	1,065	523.1	280	535.3	2.30 (2.04–2.60)	3.20 (2.82–3.63)
**Previous TB treatment history**						
New patients	56,483	30,198.7	6,606	218.8	1	1
Treatment after LTFU	1,086	560.3	303	540.8	2.53 (2.26–2.83)	2.27 (2.02–2.55)
Relapse	7,229	4066.1	982	241.5	1.14 (1.07–1.22)	1.09 (1.01–1.16)
Other previously treated patients^a^	3,124	1512.3	561	371.0	1.64 (1.50–1.79)	1.33 (1.21–1.46)
**Location of TB**						
PTB only	49,405	26,057.7	6,403	245.7	1	1
Both PTB and EPTB	18,517	10,279.8	2,049	199.3	0.83 (0.79–0.87)	0.84 (0.79–0.89)
**Number of TB notification records**						
A single record	63,387	33,919.5	7,619	224.6	1	1
Multiple records	4,535	2418.0	833	344.5	1.56 (1.46–1.67)	0.85 (0.79–0.92)
**Place of living**						
Urban	67,000	35,887.4	8,303	231.4	1	1
Rural	922	450.1	149	331.0	1.35 (1.14–1.60)	0.70 (0.59–0.83)
**Distance from home to treatment centre**						
At the same district(close)	55,620	30,846.2	4,977	161.3	1	1
Neighbouring district(far)	6,800	3169.4	1,688	532.6	3.18 (3.01–3.37)	3.24 (3.06–3.44)
Far-away district(further)	5,502	2321.9	1,787	769.6	4.51 (4.27–4.77)	4.47 (4.21–4.74)
**Chest X ray**						
Suspicious TB lesions	57,983	31,003.9	7,280	234.8	1	1
Normal	1,631	937.1	157	167.5	0.91 (0.86–0.95)	0.78 (0.66–0.93)
Unknown	1,834	951.0	216	227.1	1.04 (0.97–1.11)	0.92 (0.80–1.06)
Not done	6,474	3445.5	799	231.9	0.73 (0.63–0.86)	0.98 (0.90–1.05)
**Baseline sputum AFB smear test**						
Smear positive	22,058	11,781.9	2,837	240.8	1	1
Smear negative	36,970	19,713.1	4,389	222.6	0.95 (0.83–1.09)	1.04 (0.99–1.10)
Unknown	8,894	4842.5	1,226	253.2	0.98 (0.91–1.06)	1.41 (1.30–1.52)

^a^Composed of ‘treatment after failure patients’ and ‘other previously treated patients’ whose outcome of previous treatment was unknown or undocumented. *HR* hazard ratio, *CI* confidence interval, *LTFU* loss to follow-up, *TB* tuberculosis, *PTB* pulmonary tuberculosis, *EPTB* extra-pulmonary tuberculosis, *AFB* acid-fast bacillus.

## Discussion

In this national study of LTFU among DS-TB patients treated in the South Korean PPM, we found a higher-than-expected percentage of patients becoming LTFU when we took into account the complete picture of a patient’s interactions (or absence of interactions) with the healthcare system. The overall percentage LTFU between 2011 and 2014 was 12.2% (11.7% for single-record and 18.1% for multiple-record cases). We identified several risk factors for LTFU, such as, a greater distance between home and treatment centre, and being foreign-born. We demonstrated that attending several different TB centres during anti-TB treatment had a differential effect on LTFU depending upon the distance from home to the original treatment centre. Among the patients who initiated treatment at a nearby centre, transfer between TB centres was an independent risk factor for LTFU, whereas among patients at institutions located in districts far or even far-away from home (not in the same city, county or district), transfer out was protective.

Few studies have investigated treatment outcomes in South Korea. Those that have estimated the percentage change in LTFU as falling from 6–12% before PPM project implementation to 3% after^11,16^. However, in a nationwide study using data from KNTSS, when the outcome of ‘not evaluated’ was regarded as LTFU, percentage of LTFU in PPM institutions was higher − 9.0% (8,239/91,606) between 2012 and 2015^18^. Our results indicate that the frequency of LTFU with PPM was far higher, at 12.3% of the total cohort. It is clear, therefore, that a large proportion of LTFU cases are not officially reported in South Korea—which in turn raises issues about the current patient management system. This is particularly true given that the results of our study, which highlights the need for ongoing joined-up patient follow-up and reporting after transfer-out—something that has not been previously recognised within the healthcare administration system. This is not only a data reporting issue, but also has personal and public health implications as considerable numbers of infectious patients are likely to have not received curative treatment and may therefore have transmitted TB within their local communities.

‘Transfer-out’ can be defined in two ways- as an intermediate outcome, or an end-of-treatment outcome i.e. patients transferred to another TB centre for whom the end-of-treatment outcome is unknown by the initial centre^19^. As patients with the end-of-treatment outcome ‘transfer-out’ are highly likely to be LTFU cases, ‘transfer-out’ has been regarded as an unfavourable outcome in previous studies from other settings^20,21^. In South Korea, reporting of the end-of-treatment outcome to the original TB centre from which patients were transferred by the receiving TB centre had been limited by the Personal Information Protection Act, and not routinely performed. Therefore, in KNTSS, the term ‘transfer-out’ could both be an intermediate and an end-of-treatment outcome. In our study, 13.5% of notified TB patients were listed as intending to move from one centre to another. Another cross-sectional study at public health centres showed that the proportion transferred out was 14.3% (1554/10,834)^22^. However, in 2016, checking the status of re-registration among patients who were transferred out to other treatment centres was recommended in national guidelines for tuberculosis control, for the first time^23^. Since then, the term ‘transfer-out’ has been used as an intermediate outcome in most cases. We presumed that this thorough management might contributed to the decrease in TB burden in the late 2010s.

Although investigating the reasons for transfer-out was unfeasible in our study, one explanation for such a high proportion might be patient migration, which was a known risk factor for LTFU^24,25^. In our study, as mentioned above, the distance between home and first treatment centre modified the effects of ‘transfer between TB centres’. A substantial proportion of patients who were managed by treatment centres located ‘far-away’ might be a floating population, who live or work in another city different from their home. Although we did not investigate the second institutions after transfer-out, we speculated that a considerable proportion of transferred-out patients from this group were in fact re-registered in places closer to their home. This could result in improved family support and easier engagement with clinical care^26^. Moreover, public health centres which manage patient adherence are always located in the patient’s home district. We speculate that private hospitals far from such public health centres and patients’ home may not have the professional links in place to facilitate such collaborations.

In our study, we analysed the end-of-treatment outcomes of patients reported as ‘transfer-out’. Treatment outcomes after transfer-out have been previously reported from other settings. In two African studies, final treatment outcome was rarely conveyed back to the initial TB centre^27,28^. This is a concern given that work from Morocco suggests routinely collecting the final treatment outcome of transferred-out improves the overall treatment success rate^29^. Moreover, in a Vietnamese study, initially unrecognised patients with treatment failure or death were subsequently identified by ensuring the reporting of the transfer-out^30^. Similarly, we found that 73.9% (7,815/10,576) of TB patients reported as transfer-out, were in fact LTFU. Li et al. analysed the characteristics of TB patients in China who transferred-out, as well as the risk factors for their end-of-treatment outcome being listed as ‘not evaluated’ (indicating LTFU)^31^. They found that transfer-out to a ‘far-away’ TB centre showed the highest risk for being ‘not evaluated’.

Besides the ‘distance’ and ‘transfer-out’, we demonstrated several demographic or treatment-related risk factors for LTFU—(1) elderly TB patients, (2) foreign-born and (3) previous LTFU history. In a previous study, reasons for LTFU among TB patients managed by PPM institutions in South Korea were investigated^32^. In that study, being marginalized, adverse effects of anti-TB treatment and refusal of treatment results from lack of knowledge were the main reasons for LTFU in South Korea. Though the reasons for LTFU was not investigated in our study, we speculate that relatively high frequency of adverse effects of anti-TB medication in elderly population might be related with LTFU^33^. In addition, among Organisation for Economic Cooperation and Development (OECD) countries, South Korea showed highest relative poverty rates of elderly population, which exceeded 40% in 2016^34^. Considering that low socioeconomic status is related with poor treatment adherence and LTFU^35,36^, we presume that high LTFU rate in elderly TB patients might be attributable to elderly poverty, in part. Further studies investigating how the poverty affect treatment outcome in elderly population is required.

Similar with our results, foreign-born TB patients in South Korea showed higher rates of LTFU, than native Koreans in a previous study^37^. As some foreign-born TB patients returned to their own countries during TB treatment for visa extension or other reasons^32,37^, thorough management of these international ‘transfer-out’ by immigration authorities is required. Though insurance coverage by NHI was not significant risk factors for LTFU in that study, further large-scaled study is needed to verify the effect of insurance coverage and other socioeconomic determinants on treatment outcome in foreign-born TB patients.

Patients who had previous history of TB showed higher risk for LTFU in previous studies^16,38,39^, as in our study. Especially, those with previous LTFU showed the highest risk. Though strict directly observed therapy (DOT) is practiced only for patients with multi-drug resistant TB or cases of non-compliance, currently in South Korea^40^, DOT should be expanded for TB patients who were loss to follow-up, previously. Besides DOT, strategies to resolve the vulnerability of patients which resulted in previous LTFU such as alcoholism, lack of family support, lack of knowledge should be implemented to prevent the second LTFU.

Before the PPM project was successfully implemented nationally, monitoring treatment outcomes with KNTSS was unfeasible for the following reasons: (1) the data included in the KNTSS are mainly used to capture mandatory TB notifications, which limits their use in monitoring treatment outcome, (2) after notification, patients’ treatment outcome data are not routinely updated, (3) inter-hospital transfer of TB records was unavailable in the KNTSS for the reason mentioned above. Our study has demonstrated the limitations of conventional KNTSS for monitoring. We propose that monitoring and evaluation of national TB control programmes via the PPM project, with its country-wide reach and ability to provide a complete picture of TB healthcare encounters, is a viable alternative^41^.

Our study has some limitations. (1) There may be a selection bias resulting from censoring a substantial proportion of TB patients (who received treatment for 301 days or more and whose outcome was reported as success with insufficient treatment duration). (2) As this was a study with multiple exposures, some of them may in fact be on the causal pathway between others and the outcome. This could result in biased effect estimates. (3) We could not identify the reasons for LTFU and socioeconomic or environmental vulnerability of patients, as that information is not collected in KNTSS.

In conclusion, by examining the complete picture of a patient’s interactions with healthcare during their treatment for TB, we have identified a higher-than-expected rate of LTFU among PPM patients in South Korea—particularly those not managed at treatment centres near to their home. Our work highlights what needs to be done within the PPM project to improve the validity of outcome reporting and reduce LTFU. This has implications for other settings with similar models of healthcare provision, as well as other infectious diseases where surveillance is a critical tool^42^.

## Methods

### Study population

All TB patients in South Korea are reported to the KNTSS^43^. Cases notified between 1 August 2011 and 31 July 2014 in public–private mix (PPM) institutions were extracted from the database on 31 May 2015, thus including at least 10 months of follow-up for each patient. Exclusion criteria were as follows—multidrug-resistant TB, presence of rifampicin or isoniazid mono-resistance, DS-TB treated without rifampicin, TB involving the spinal, skeletal, or central nervous system, change of diagnosis, or data errors.

### Merging, and reclassification of treatment outcomes

The process of merging and reclassifying the 10 raw outcomes recorded on KNTSS (cure, completion, failure, LTFU, transfer-out, TB-related death, TB-unrelated death, still on treatment, diagnosis change and others) into six integrated outcomes by an operational definition (treatment success, failure, LTFU, still on treatment, death, and others) is described in the Supplementary Note. In cases of relapse, only the first record was included. Treatment outcomes—cure, completion, LTFU, failure, and death—within KNTSS were defined according to the WHO criteria^19^.

### Exposure variables

Demographic characteristics, results of microbiological examination, details of anti-TB regimens, and final treatment outcomes were included in the KNTSS dataset. All patients were classified into five age groups (< 20, 20–34, 35–49, 50–64, ≥ 65). Distance from home to the treatment centre was calculated indirectly based on hospital location and the district where the patients lived. The distance was classified into instances where the hospital and patient’s residence were within the same municipal level divisions (district, city, or county) (close), in different district, city or county but located within the same large administrative divisions (province or metropolitan city) (far) or within the different large administrative divisions (far-away). Considering that the average area of district, city and county in South Korea is 49.8 km^2^, 539.5 km^2^ and 669.3 km^2^, respectively, the estimated geographical distance of ‘close’ would range from several kilometres up to approximately 50 km. In addition, as the average area of a metropolitan city and province in South Korea is 736.2 km^2^ and 11,813.9 km^2^ respectively, we can speculate that the distance representing ‘far-away’ would be considerably more than 50 km, with a maximum of several hundred kilometres. The classification of ‘far’ would range between that of ‘close’ and ‘far-away’.

Patients were also classified into four categories by history of previous treatment for TB (types of registration): new, treatment after LTFU, relapse, and other previously treated patients. The category ‘other previously treated patients’ was composed of ‘treatment after failure patients’ and ‘other previously treated patients’ which were defined according to the WHO criteria^19^.

Having multiple records before LTFU- indicating that the patient had transferred between TB centres before the final treatment outcome was reported—was also assessed as a risk factor of interest.

### Statistical analysis

The percentage of patients LTFU was calculated and then risk factors for LTFU were investigated in a time-to-event model with events of competing risk, where ‘LTFU’ was the outcome of interest, ‘death’, ‘failure’ and ‘treatment success’ the outcomes with competing risk, and other outcomes were censored. To avoid bias associated with an extended treatment duration, which increases the risk of LTFU, the maximum follow-up period of all cases was limited to 300 days. Cases with outcomes reported after (>) 300 days were reclassified as ‘still on treatment’ and censored in the analysis. Univariable and multivariable competing risks analyses were used to assess the association between LTFU and demographic, clinical, and hospital-specific variables and performed with the Fine and Gray method. A sensitivity analysis restricted the study population to only patients with pulmonary TB. Statistical analyses were conducted with R v.3.5.2 (R foundation for Statistical Computing, Vienna, Austria).

### Ethics approval

The study protocol was approved by the Institutional Review Board of Incheon St. Mary’s Hospital, Korea (IRB No: OC14RCSI0149) and the need for informed consent was waived given the retrospective nature of the study. All patients' records were previously anonymised to ensure patient confidentiality. All methods were performed in accordance with the relevant guidelines and regulations.

## Supplementary Information

Supplementary Information.

## Data Availability

Korea Disease Control and Prevention Agency (KDCA) owns all datasets. The data used in the current study are available only after the permission from the KDCA in advance.
