# Author Correction: Hidden loss to follow-up among tuberculosis patients managed by public–private mix institutions in South Korea

**DOI:** 10.1038/s41598-023-30025-z

**Published:** 2023-02-21

**Authors:** Hyung Woo Kim, Sohee Park, Jinsoo Min, Jiyu Sun, Ah Young Shin, Jick Hwan Ha, Jae Seuk Park, Sung-Soon Lee, Marc Lipman, Ibrahim Abubakar, Helen R. Stagg, Ju Sang Kim

**Affiliations:** 1 Division of Pulmonary and Critical Care Medicine, Department of Internal Medicine, Incheon St. Mary’s Hospital, College of Medicine, The Catholic University of Korea, Seoul, Republic of Korea; 2 Institute of Health Services Research, Yonsei University, Seoul, Republic of Korea; 3 Department of Biostatistics, Graduate School of Public Health, Yonsei University, Seoul, Republic of Korea; 4 Division of Pulmonary and Critical Care Medicine, Department of Internal Medicine, Seoul St. Mary’s Hospital, College of Medicine, The Catholic University of Korea, Seoul, Republic of Korea; 5 Division of Biostatistics, Department of Biomedical Systems Informatics, Yonsei University College of Medicine, Seoul, Republic of Korea; 6 Division of Pulmonary Medicine, Department of Internal Medicine, Dankook University College of Medicine, Cheonan, Republic of Korea; 7 Division of Pulmonary and Critical Care Medicine, Department of Internal Medicine, Ilsan Paik Hospital, Inje University College of Medicine, Goyang, Republic of Korea; 8 UCL-TB, University College London, London, UK; 9 Division of Medicine, UCL Respiratory, University College London, London, UK; 10 Royal Free London NHS Foundation Trust, London, UK; 11 Institute for Global Health, University College London, London, UK; 12 Usher Institute, The University of Edinburgh, Edinburgh, UK

Correction to: *Scientific Reports* 10.1038/s41598-022-16441-7, published online 20 July 2022

The original version of this Article contained an error in the Author information section.

“These authors contributed equally: Hyung Woo Kim, Sohee Park, Helen R. Stagg and Ju Sang Kim.”

now reads:

“These authors contributed equally: Hyung Woo Kim and Sohee Park.

These authors jointly supervised this work: Helen R. Stagg and Ju Sang Kim.”

The original Article has been corrected.

