# Supplementary material for: Hidden loss to follow-up among tuberculosis patients managed by public–private mix institutions in South Korea
Source: Sci Rep. 2022 Jul 20;12:12362. doi: 10.1038/s41598-022-16441-7 (PMC9300674; doi:10.1038/s41598-022-16441-7)
Supplement: Supplementary file 1 — Supplementary Information. [file 41598_2022_16441_MOESM1_ESM.pdf]

Subdivision of outcome, 'transfer-out'

1) Records were initially divided into those whose outcomes were reported as 'transfer-out' and those who did not. The records reported as 'transfer-out' were subdivided into 3 groups by the re-registration status - 'No further registration', 'Re-registration within (≤) 60 days', 'Re-registration after (>) 60 days'.

2) Cases with 'No further registration' were checked to ensure that they had at least 60 days of follow-up after transfer-out. If they had insufficient follow-up time, they were reclassified into 'still on treatment'.

Cleaning and reclassification process of single record patients

1) If a favorable outcome (cure or completion) occurred before 5.5 months of follow-up (< 166 days) had elapsed since the first registration date, these outcomes were rejected as patients had taken insufficient treatment. These cases were regarded as that the outcome did not occur (censored), statistically the same concept as 'still on treatment'. So, these outcomes were reclassified into 'still on treatment'.

2) Patients who had any outcome recorded more than 10 months (>300 days) after the first registration date were reclassified as 'still on treatment' at this time point in order to ensure a consistent maximum follow up time. Follow-up data after this time point were not included.

3) For patients who did not transfer-out and who did not meet either of the two criteria above, their LTFU status was taken from the Korean National TB Surveillance System.

4) To define loss to follow-up (LTFU) for the transfer-out patients, we checked to ensure that patients had at least 60 days of follow-up after transfer-out. If they did, then the patient was classified as LTFU. If the patient did not have enough follow-up, point 2 in the section above (*Subdivision of outcome, 'transfer-out'*) applied.

Cleaning, merging and reclassification process of multiple records patients

After identifying multiple records for the same patients, we arranged all the records in chronological order, and then redefined the registration in the following way.

1) Favorable outcomes were detected for each patient. If they occurred before 5.5 months of follow-up (< 166 days) of follow-up, they were ignored. The latest remaining favorable outcome was then selected. Any records with outcomes after the date of this favorable outcome were deleted.

2) Any records with outcomes reported more than 10 months (>300 days) after the first registration date were classified into 'still on treatment' at this time point in order to ensure a consistent maximum follow up time.

3) Potential records with LTFU were identified (‘LTFU’, ‘Not re-registered case after transfer-out’, ‘Re-registration after (>) 60 days’). For each potential instance of LTFU, the amount of time out of contact with healthcare services was determined. If time between the date of LTFU and next registered date was less than 61 days, the record was reclassified as not true LTFU. The earliest event of true LTFU was then selected.

4) Deaths were detected for each patient of either kind – TB related or unrelated. The earliest event of death was selected.

5) After redefining the treatment outcome by aforementioned way, we combined all the records into a single true outcome per one patient with an associated date. First, we identified only patients with LTFU (with its associated date). Secondly, if the favorable outcome existed before LTFU, we categorized it as favorable outcome (with its associated date). Thirdly, if neither a favorable outcome nor LTFU was identified and death happened, or death occurred before LTFU or favorable outcome, we categorized the patient as died (with its associated date). Lastly, patients without any outcomes at this stage were then classified according to any other last outcome recorded.

6) In addition, only the first registration date was also recorded per patient.

7) The final step was the extraction of demographic and clinical data from only the first episode i.e. patient status at baseline.
